# Quaking and *miR-155* interactions in inflammation and leukemogenesis

**DOI:** 10.18632/oncotarget.5248

**Published:** 2015-08-24

**Authors:** Esmerina Tili, Marcela Chiabai, Dario Palmieri, Melissa Brown, Ri Cui, Cecilia Fernandes, Tim Richmond, Taewan Kim, Tyler Sheetz, Hui-Lung Sun, Alessandro Lagana, Dario Veneziano, Stefano Volinia, Laura Rassenti, Thomas Kipps, Hamdy Awad, Jean-Jacques Michaille, Carlo M. Croce

**Affiliations:** ^1^ Department of Anesthesiology, Wexner Medical Center, The Ohio State University, Columbus, OH, USA; ^2^ Department of Molecular Virology, Immunology and Medical Genetics, The Ohio State University, Wexner Medical Center and Comprehensive Cancer Center, Columbus, OH, USA; ^3^ Department of Molecular and Cellular Oncology, The University of Texas MD Anderson Cancer Center, Houston, TX, USA; ^4^ Department of Genetics and Genomic Sciences, Icahn School of Medicine at Mount Sinai, New York, NY, USA; ^5^ University of Ferrara, Department of Morphology, Surgery and Experimental Medicine, Ferrara, Italy; ^6^ CLL Research Consortium, Moores UCSD Cancer Center, La Jolla, CA, USA; ^7^ BioPerox-IL, UB-INSERM IFR #100, Université de Bourgogne, Faculté Gabriel, Gabriel, Dijon, France

**Keywords:** miR-155, CLL, inflammation, QKI, glioblastoma

## Abstract

*Quaking* (*QKI*) is a tumor-suppressor gene encoding a conserved RNA-binding protein, whose expression is downregulated in several solid tumors. Here we report that QKI plays an important role in the immune response and suppression of leukemogenesis. We show that the expression of *Qki* is reduced in lipopolysaccharide (LPS)-challenged macrophages, suggesting that *Qki* is a key regulator of LPS signaling pathway. Furthermore, LPS-induced downregulation of Qki expression is *miR-155*-dependent. *Qki* overexpression impairs LPS-induced phosphorylation of JNK and particularly p38 MAPKs, in addition to increasing the production of anti-inflammatory cytokine IL-10. In contrast, *Qki* ablation decreases *Fas* expression and the rate of Caspase3/7 activity, while increasing the levels of IL-1α, IL-1β and IL-6, and p38 phosphorylation. Similarly, the p38 pathway is also a target of QKI activity in chronic lymphocytic leukemia (CLL)-derived MEC2 cells. Finally, B-CLL patients show lower levels of *QKI* expression compared with B cells from healthy donor, and *Qki* is similarily downregulated with the progression of leukemia in *Eμ-miR*-155 transgenic mice. Altogether, these data implicate QKI in the pathophysiology of inflammation and oncogenesis where *miR-155* is involved.

## INTRODUCTION

*miR-155* is an oncogenic pro-inflammatory microRNA (miRNA) that is up-regulated in a number of solid tumors and liquid malignancies [1–3]. High levels of *miR-155* often correlate with a poor prognosis [4–5]. Targeted expression of *miR-155* in B cells results in pre-B cell acute leukemia/high-grade lymphoma [6]. Furthermore, overexpression of *miR-155* in lymphoid tissues results in disseminated lymphoma characterized by a clonal, transplantable pre-B-cell population of neoplastic lymphocytes “addicted” to *miR-155*-activity [7]. In hematopoietic cells, the expression of *miR-155* is controlled by several immune signals [1–3]. Thus, LPS induces *miR-155* expression in macrophage/monocytic cell lines of both mouse and human origin [8–9]. The oncogenic and pro-inflammatory effects of *miR-155* have been attributed at least in part to its targeting of many transcripts encoding tumor suppressors and/or anti-inflammatory factors, especially *Ship1* [10–12], *Socs1* [13].

Quaking (*QKI*, KH domain containing, RNA binding) is a member of the signal transduction and activation of RNA (STAR) family of RNA-binding proteins. Three major QKI isoforms (QKI-5, QKI-6 and QKI-7), each with a specific carboxy-terminal end, are produced through alternative splicing both in mouse and human [14]. QKI-5 contains a nuclear localization signal, is predominantly localized in the nucleus, and is most likely to function in pre-mRNA splicing or RNA retention. In contrast, QKI-6 is localized in both the nucleus and the cytoplasm, while QKI-7, exclusively cytoplasmic, is pro-apoptotic [14–16]. *QKI* behaves as a tumor suppressor gene (TSG) in glioblastoma multiforme (GBM) [17]. QKI, under the direct control of p53 in GBM cells [17], is also downregulated in gastric and colon cancers [18–19]. In GBM cells, QKI can associate with and stabilize *miR-20a*, thus, increasing *miR-20a* down-regulatory effects on TGF-β receptor type II (TGF-βR2), whose activity is oncogenic in gliomagenesis [17].

As *QKI* is the first most probable target of *miR-155* in apes and human (targetscan.org), and that the expression of *miR-155* is upregulated in glioblastomas [20–21], the above-mentioned facts suggested that *miR-155* might carry out its oncogenic and pro-inflammatory functions at least in part by targeting *QKI*. In the present study, we show that (i) *QKI* is indeed a target of *miR-155* in B cells; (ii) the expression of QKI is lower in B-cells from CLL patients compare to B cells from healthy donors, and acts as TSG also in CLL; (iii) Qki is a target of LPS signaling, and its expression is downregulated following LPS challenge of macrophages; (iv) Qki modulates downstream *LPS* signaling in return; particularly p38 MAPK activation and IL-10 production, thus presenting with anti-inflammatory properties. We propose that *miR-155* and *QKI* form a critical regulatory component downstream of TLR4 in target hematopoietic cells, and that *miR-155* exerts its pro-inflammatory and oncogenic activities at least in part through the downregulation of *QKI* expression.

## RESULTS

### *QKI* is downregulated at the onset of the innate immune response to LPS

**Figure 1:**
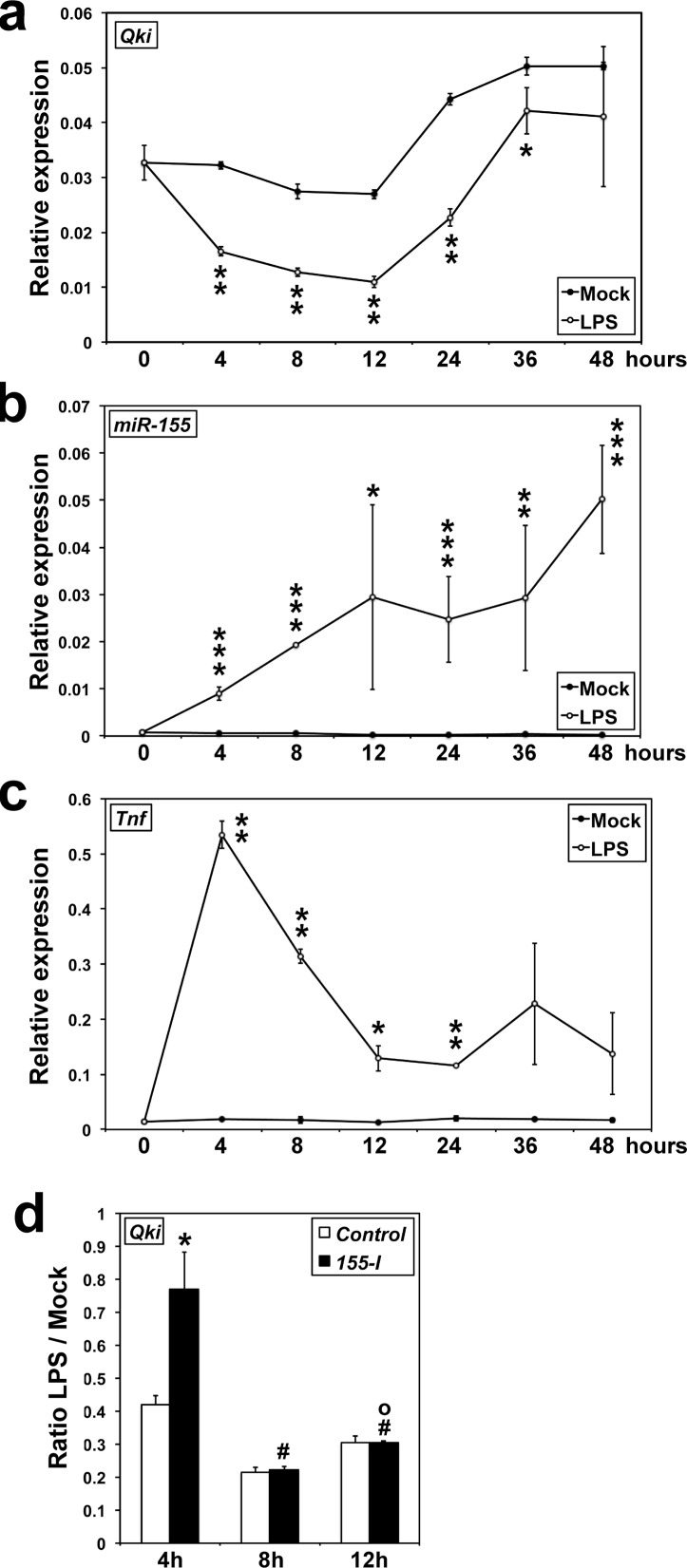
*QKI* expression is downregulated at the onset of LPS challenge **A.–C.** *Qki, miR-155* and *Tnf* levels in RAW-264.7 cells were measured by qRT-PCR (*n* = 3); **A.**: *P = 0.0636; **P < 0.000345. **B.**: *P = 0.06177; **P = 0.03106; ***P < 0.00168. **C.**: *P = 0.0124; **P < 0.000786. **D.** RAW-264.7 cells transfected with either a *Control-RNA* (Control) or an antisense *miR-155* inhibitory RNA (*155-I*) were challenged with LPS 24 hours later. *Qki* transcripts levels were measured by qRT-PCR (*n* = 3). *miR-*155-I* different from Control, *P* < 0.037. ^#^ 8-hours different from 4-hours, *P* < 0.0052. ^o^12-hours different from 8-hours, *P* < 0.0044.

We have previously shown that the expression of *miR-155* is upregulated in mouse RAW-264.7 macrophages treated with LPS [8]. As Qki is a potential target of *miR-155*, we monitored the expression of *Qki*, using a probe spanning exons 4 and 5 that recognizes all *Qki* isoforms, as well as of *miR-155* and *Tnf* in mouse RAW-264.7 macrophages following LPS stimulation. *Qki* expression decreased 2-fold within 4 hours, while the expression of *miR-155* as well as that of *Tnf*, both immediate downstream targets of LPS signaling, increased significantly (Figure 1A–1C). Beyond 12 hours, *Qki* transcripts progressively returned to their initial level at 2-days, while *miR-155* level kept increasing. Of note, the expression of *Qki* in untreated cells subsequently increased above its initial level, an effect delayed by roughly 12 hours following LPS challenge (Figure 1A). This increase was probably related to the surge of cell density, as previously reported in HT29 colon cancer cells [22]. Semi-quantitative RT-PCR analysis using oligonucleotides designed to specifically recognize each of *Qki* isoforms, showed that both *Qki-5* and *Qki-6* are expressed in RAW-264.7 cells and their expression is downregulated at the beginning of LPS challenge (data not shown), further confirming our hypothesis. On Western blots, Qki levels also decreased significantly only for a short period of time (data not shown), suggesting that Qki might be a critical component of LPS signaling, potentially involved in preventing the initiation of an unnecessary innate immune response, assuring the robustness of the response at its onset, and resolving and terminating the response later on. Our pan-Qki antibody detected one Qki isoform only, and QKI-5 being the main isoform expressed in hematopoietic cells [23], this isoform is likely to represent Qki-5.

Accordingly, an antisense *miR-155* inhibitory RNA (*155*-*I*) protected *Qki* transcript from downregulation only during the first 4 hours of LPS challenge, while remaining without effects later on (Figure 1D). Altogether, the above results suggest that, in RAW-264.7 macrophages, *Qki* transcripts are potentially targets of *miR-155* at the onset of LPS signaling only.

### *QKI* is a direct target of *miR-155* in U937 cells

**Figure 2:**
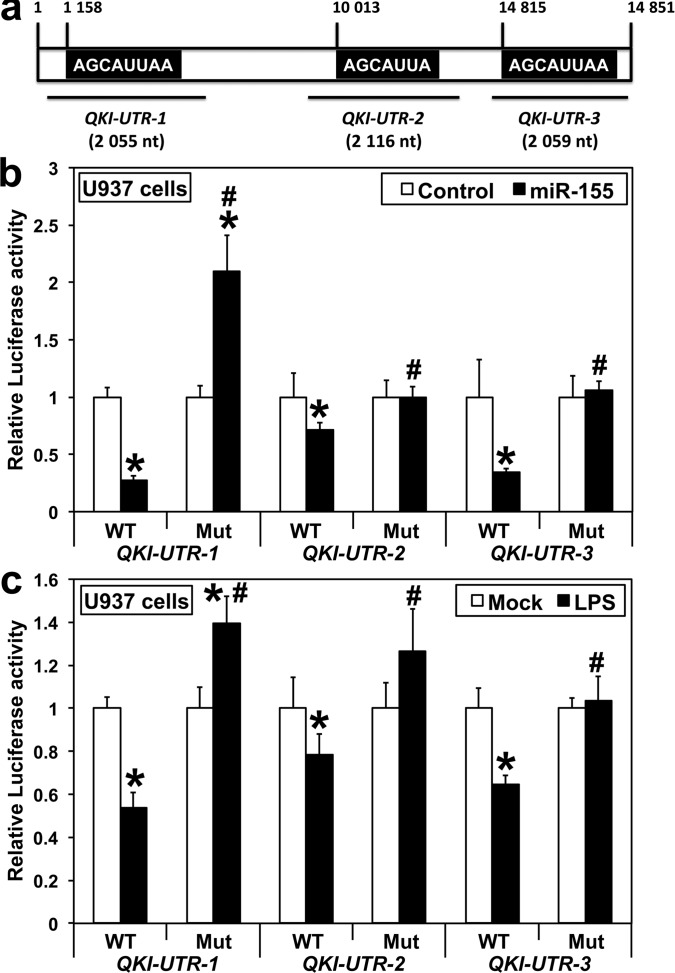
*QKI* is a direct target of *miR-155* in human U937 monocytes **A.** Schematic (not-to-scale) representation of human *QKI-3′-UTR*. The sequences of *miR-155* consensus target sites (highlighted) present in the three *QKI*-3′-UTR *Luciferase* reporter constructs are shown. **B.** Cells were co-transfected with either *QKI-UTR-1, QKI-UTR-2* or *QKI-UTR-3*, each containing either a wild type (WT) or a mutated (Mut) *miR-155* target site, along with either *pre-miR-Control* (Control) or *pre-miR-155* (*n* = 12). **C.** Cells were transfected with either WT/Mut *QKI-UTR-1, QKI-UTR-2* or *QKI-UTR-3* 24 hours before LPS challenge (*n* = 12). Values for *pre-miR-Control* **B.** and Mock **C.** were arbitrarily set to 1. Assays were performed three times in quadruplicates 48 hours after transfection. **B.** *miR-155 different from Control, *P* < 0.00052. **C.** *LPS different from Mock **C.**, *P* < 0.0022. **B.**, **C.** ^#^Mutant different from WT, *P* < 1×10^−6^.

Human *QKI*-3′-UTR contains three putative *miR-155* target sites, the first one being conserved among eutherian mammals but absent in muridae and in some human isoforms, the second highly conserved across vertebrates, and the third one primate-specific (targetscan.org). To determine whether *miR-155* could directly target *QKI* transcripts, we prepared three *Luciferase* reporter constructs from human *QKI*-3′-UTR, each containing one *miR-155* target site (Figure 2A). A *miR-155* mimic co-transfected in U937 cells with each of these constructs decreased the Luciferase activity produced from the constructs containing a WT, but not a mutant, *miR-155* site (Figure 2B). Finally, the Luciferase activity also decreased when U937 cells were first transfected with constructs containing the WT, but not the mutant, *miR-155* site and then challenged with LPS (Figure 2C), giving a further evidence that *QKI* downregulation at the onset of the innate immune response to LPS results from its direct targeting by *miR-155*. Intriguingly, mutating the *miR-155* target site of *QKI-UTR-1* increased the Luciferase activity produced following *miR-155* transfection beyond that of the Control (Figure 2B, 2C). This may possibly result from *miR-155* targeting immune factor(s) that bind to this particular region of *Qki*-3′-UTR, given that the stability of several immune transcripts is regulated by the fixation of RNA binding proteins on their 3′-UTR, and that we observed a similar phenomenon in RAW264.7 macrophages, but not in HEK-293 kidney cells (not shown).

### Qki regulates cytokine production and MAPK phosphorylation

**Figure 3:**
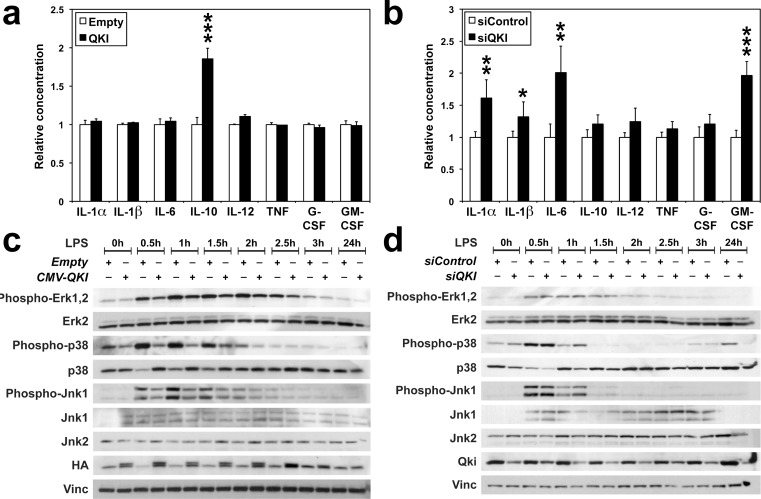
QKI impairs Mapk activation and interleukin production **(A-D)** RAW-264.7 cells were challenged with LPS 24 hours following transfection with either an empty *CMV* vector (Empty) or a construct expressing human *QKI-5* (QKI) **(A, C),** or with either a *control-siRNA* (*siControl*) or *siQKI* **(B, D). A.**, **B.** Supernatants harvested 24 hours after LPS treatment were analyzed by ELISA assay for the indicated cytokines. Values were normalized to *Control*. *P = 0.069; **P < 0.019; ***P < 0.001. **C.-D.** Phosphorylation of Erk1/Erk2, Jnk1/Sapk and p38 Mapk following LPS stimulation was followed on Western blots. Stripes in **C.** and **D.** come from three different gels prepared from the same extracts. HA: HA-tagged QKI. Vinculin (Vinc) was used as a loading control.

*Qki* being a target of LPS signaling suggested that Qki itself might be a modulator of the innate immune response. We thus transfected RAW-246.7 cells with a construct expressing *QKI-5* (hereafter referred to as *QKI*), chosen because: (i) it is the only QKI isoform that contains a nuclear localization signal and functions in pre-miRNA splicing and/or RNA retention [15], thus being the most likely isoform to impact the stability and processing of immune transcripts; and (ii) QKI-5 is the main isoform expressed in hematopoietic progenitors and differentiated cells [23], and our experiments in Figure 1 showed that it is well expressed in RAW-264.7 cells. Indeed, the over-expression of *QKI-5* doubled the level of anti-inflammatory IL-10 in cell supernatant (Figure 3A), 24 hours after LPS challenge. In contrast, targeting *Qki* transcripts with small interfering RNAs (*siQKI)*, lead to higher levels of inflammatory IL-1α, IL-1β, IL-6 and GM-CSF (Figure 3B). The fact that QKI enhanced IL-10 production, suggests that its downregulation during the first hours of LPS challenge is a prerequisite for the immune response to reach the optimal threshold level of activation. As LPS binding to TLR4 receptors triggers the downstream activation of ERK, JNK and p38 MAPK pathways in responding cells [24], we then analyzed the effects of QKI on the three above pathways. Overexpressing *QKI* impaired the phosphorylation of p38 and Jnk1/Sapk MAPKs, possibly also slightly the phosphorylation of Erk1, 2, during the two first hours of LPS challenge (Figure 3C). In contrast, *siQKI* respectively increased p38 phosphorylation by 47% at 0.5-hour (*P* = 0.0011) and by 177% at 1-hour (*P* = 0.0789) (Figure 3D), as determined by scanning the bands on blots representing three different biological replicates. Noteworthy, *QKI* overexpression sharply increased the level of total p38, however only residual amount of this kinase was phosphorylated (Figure 3C). As the p38 MAPK pathway is central to the stabilization of many mRNAs encoding factors implicated in the innate and adaptive immune responses, including cytokines [24–26], these results further confirm our hypothesis that the early down-regulation of *Qki* by LPS signaling pathway is needed for mounting an effective immune response.

### *miR-155* also targets *QKI* in human leukemic B lymphocytes

**Figure 4:**
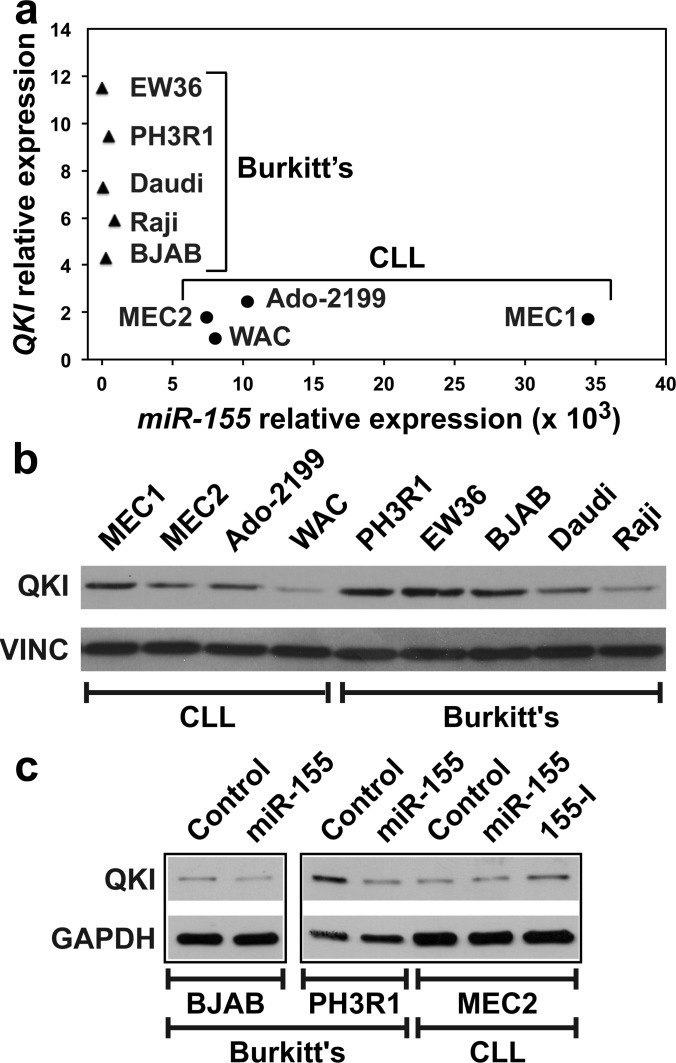
Burkitt's and CLL cell lines display different levels of *QKI* and *miR-155* expression **A.** *QKI* and *miR-155* levels were measured by qRT-PCR in five Burkitt's lymphoma cell lines (filled triangles) and in four CLL-derived cell lines (filled circles). **B.** QKI protein levels in the same cells as in **A.**. **C.** Indicated cells were transfected with either a Control RNA or *miR-155*. MEC2 cells were also transfected with *155-I*. Western blot was performed 48 hours following transfection. Panels are from the same blot.

The expression of *miR-155* is high in B-CLL-derived cell lines as well as in CLL patients [27–30], but low in B-cell Burkitt's lymphomas [31]. We found that *QKI* and *miR-155* expression well discriminate Burkitt's cell lines, all with lower *miR-155* and higher *QKI* levels, from CLL cell lines, all with higher *miR-155* but lower *QKI* levels (Figure 4A). Accordingly, QKI protein levels in MEC2, Ado-2199 and WAC CLL cells were lower than those in PH3R1, EW36 and BJAB cells (Figure 4B). However, QKI levels in MEC1, Daudi and Raji cells suggest that QKI expression at the protein level is also regulated by other, non-*miR-155*-dependent mechanisms. Nevertheless, *miR-155* mimic reduced *QKI* expression in BJAB and PH3R1 Burkitt's cells, but not in MEC2 CLL cells (Figure 4C). In contrast, transfecting MEC2 cells with *155-I* slightly but significantly increased QKI levels (Figure 4C). Altogether, these results indicate that *QKI* is also a target of *miR-155* in B cells.

### QKI regulates *FAS* expression and Caspases 3/7 activity in B cells

**Figure 5:**
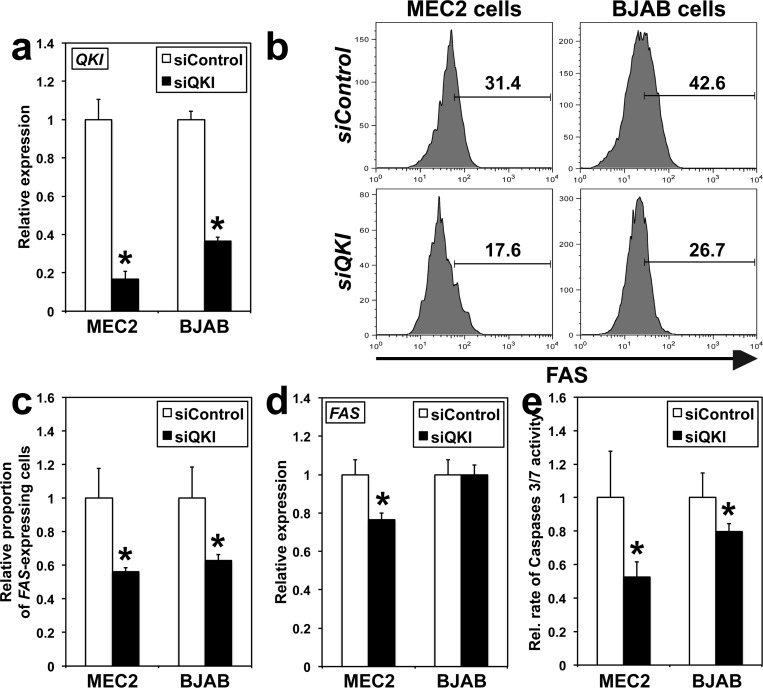
QKI effects on *FAS* expression and Caspases 3/7 activity in BJAB and MEC2 cells Analyses were conducted 48 hours following transfection. **A.** *QKI* transcripts levels were determined using qRT-PCR. *siQKI different from *siControl, P* < 0.0003 (*n* = 3). **B.** Representative histograms of FAS staining on MEC2 and BJAB cells as determined by flow cytometry (*n* = 3). **C.** Graphical presentation of triplicate results of flow cytometry run in **B.**. *P < 0.026 (*n* = 3). **D.** *FAS* expression was analyzed using qRT-PCR. *P < 0.01. **E.** The relative rate of Caspases 3/7 activity was measured in cells transfected either with *siQKI* or *siControl*. *P < 0.031. In **A.** and **C.-E.**, values for *siControl* were arbitrarily set to 1.

A bioinformatics analysis showed that the 3′-UTR of *FAS* transcripts contains the consensus sequence for QKI binding [32]. *siQKI*, functional in both MEC2 and BJAB cell lines (Figure 5A), significantly reduced FAS levels and the percentage of *Fas*-expressing cells in both cell lines (Figure 5B, 5C). Furthermore, *siQKI* decreased *FAS* transcript levels in MEC2 but not BJAB cells (Figure 5D), suggesting that different molecular mechanisms control *FAS* expression in these two types of hematological malignancies. Finally, *siQKI* decreased the activity of Caspases 3/7 in both cell lines (Figure 5E), suggesting that QKI might act as a TSG also in B cells.

### QKI is implicated in lymphocyte function and homeostasis

We then analyzed transcriptome modifications induced by *siQKI* in MEC2 and BJAB cells using Affymetrix arrays (MIAME: E-MTAB-2375) and Ingenuity software. Expectedly, pathways implicated in the regulation of lymphocyte differentiation, proliferation, function and/or signaling were affected in both cell lines (not shown). However, *siQKI* had also cell specific effects (Tables 1 and 2). Of interest, the p38 pathway, and particularly *IRAK3* expression were affected by *siQKI* in MEC2 cells (Table 1). This result and those of Figures 3C, 3D, suggest that p38 pathway is regulated by QKI in different types of hematopoietic cells. Of note, 30% of the 100 transcripts, most significantly affected by *siQKI* in MEC2 cells were predicted targets of *miR-155* (Supplementary Table 2), versus 23% in BJAB cells (Supplementary Table 3). Thus, variations in QKI levels in B cells (Figure 4) are likely to be instrumental in both Burkitt's and CLL cell transformation.

**Table 1:** The twenty most significant pathways affected by *siQI* transfection in MEC2 CLL cells

Rank	Ingenuity Canonical Pathways	*P*-value	Transcripts
1	Role of Oct4 in Mammalian Embryonic Stem Cell Pluripotency	1.05E-03	*FAM208A, RARA, MEF2A, IGF2BP1*
2	T Helper Cell Differentiation	5.13E-03	***TGFBR1, CD80**, HLA-DOB*, CXCR5
3	Notch Signaling	6.46E-03	*ADAM17, DTX3, JAG1*
4	Cardiac Hypertrophy Signaling	7.41E-03	*ROCK2, **GNAS, TGFBR1**, IGF1, IRS1, SOS1, MEF2A*
5	Role of NFAT in Regulation of the Immune Response	7.59E-03	***GNAS, CD80**, SOS1, MEF2A, HLA-DOB, ITPR1*
6	PPARα/RXRα Activation	8.71E-03	***GNAS***, ***TGFBR1****, GPD2, IRS1, SOS1, ACVR2B*
7	TGF-β Signaling	9.77E-03	***TGFBR1****, SOS1, ACVR2B,* SMAD5
8	Role of NFAT in Cardiac Hypertrophy	1.02E-02	***GNAS***, ***TGFBR1****, IGF1, SOS1, MEF2A, ITPR1*
9	Chronic Myeloid Leukemia Signaling	1.48E-02	***TGFBR1****, CTBP2, SOS1, E2F5*
10	IGF-1 Signaling	1.62E-02	*NEDD4, IGF1,* ***IRS1****, SOS1*
11	Cholecystokinin/Gastrin-mediated Signaling	1.86E-02	*ROCK2, SOS1, MEF2A, ITPR1*
12	β-alanine Degradation I	2.00E-02	*ABAT*
13	Glycine Degradation (Creatine Biosynthesis)	2.00E-02	*GATM*
14	Antiproliferative Role of TOB in T Cell Signaling	2.75E-02	***TGFBR1****, TWSG1*
15	Glycerol-3-phosphate Shuttle	2.95E-02	*GPD2*
16	4-aminobutyrate Degradation I	2.95E-02	*ABAT*
17	GDNF Family Ligand-Receptor Interactions	3.09E-02	***IRS1****, SOS1, ITPR1*
18	p38 MAPK Signaling	3.09E-02	***TGFBR1****, MAPT, MEF2A, IRAK3*
19	IL-4 Signaling	3.80E-02	*IRS1, SOS1, HLA-DOB*
20	B Cell Development	4.07E-02	***CD80****, HLA-DOB*

Transcripts whose levels also changed in BJAB Burkitt's cells are given in bold letters.

**Table 2:** The twenty most significant pathways affected by *siQI* transfection in BJAB Burkitt's cells

Rank	Ingenuity Canonical Pathways	*P*-value	Transcripts
1	Dendritic Cell Maturation	2.69E-05	*COL1A1,* ***CD80****, PIK3CG, IL32, FSCN1, AKT3, LTB, IL1B, TLR3, CCR7*
2	Lymphotoxin β Receptor Signaling	3.02E-04	*VCAM1, PIK3CG, AKT3, LTB, TRAF1*
3	Crosstalk between Dendritic Cells and Natural Killer Cells	4.57E-04	***CD80****, CD69, FSCN1, LTB, TLR3, CCR7*
4	NF-κB Signaling	4.90E-04	***TGFBR1****, HDAC2, PIK3CG, IGF1R, AKT3, IL1B, TLR3, TNFRSF17*
5	Role of Macrophages, Fibroblasts and Endothelial Cells in Rheumatoid Arthritis	5.50E-04	*VCAM1, C5AR1, PIK3CG, IL32, RAC1, AKT3, LTB, IL1B, TLR3, WNT5A, TRAF1*
6	Docosahexaenoic Acid (DHA) Signaling	8.51E-04	*PIK3CG, AKT3, IL1B, APP*
7	Hepatic Fibrosis / Hepatic Stellate Cell Activation	8.51E-04	*COL1A1, VCAM1,* ***TGFBR1****, TIMP1, IGF1R, IL1B, CCR7*
8	Role of Tissue Factor in Cancer	1.38E-03	*PIK3CG, EGR1, RAC1, ITGA6, AKT3, IL1B*
9	Altered T Cell and B Cell Signaling in Rheumatoid Arthritis	2.51E-03	***CD80****, LTB, IL1B, TLR3, TNFRSF17*
10	RAR Activation	3.09E-03	***GNAS****, DHRS9, PIK3CG, RAC1, AKT3,* ***SMAD5***, *RXRA*
11	Communication between Innate and Adaptive Immune Cells	3.24E-03	***CD80****, IL1B, TLR3, CCR7, TNFRSF17*
12	Human Embryonic Stem Cell Pluripotency	3.55E-03	*GNAS,* ***TGFBR1****, PIK3CG, AKT3,* ***SMAD5***, *WNT5A*
13	Regulation of the Epithelial-Mesenchymal Transition Pathway	3.80E-03	***TGFBR1****, PIK3CG, EGR1, ZEB2, PARD6B, AKT3, WNT5A*
14	Role of Pattern Recognition Receptors in Recognition of Bacteria and Viruses	4.07E-03	*CLEC7A, C5AR1, PIK3CG, IL1B, TLR3*
15	CXCR4 Signaling	6.76E-03	*MYL12A,* ***GNAS****, PIK3CG, EGR1, RAC1, AKT3*
16	Sphingosine-1-phosphate Signaling	7.24E-03	***GNAS****, PIK3CG, RAC1, AKT3, ASAH1*
17	Role of NANOG in Mammalian Embryonic Stem Cell Pluripotency	7.41E-03	*PIK3CG, AKT3,* ***SMAD5****, WNT5A, TCL1A*
18	Small Cell Lung Cancer Signaling	7.59E-03	*PIK3CG, AKT3, RXRA, TRAF1*
19	NF-κB Activation by Viruses	8.71E-03	*PIK3CG, ITGA6, AKT3,* CXCR5
20	PTEN Signaling	9.33E-03	***TGFBR1****, PIK3CG, IGF1R, RAC1, AKT3*

Transcripts whose levels also changed in MEC2 CLL cells are given in bold letters.

### *QKI* is downregulated in B cells of CLL patients

**Figure 6:**
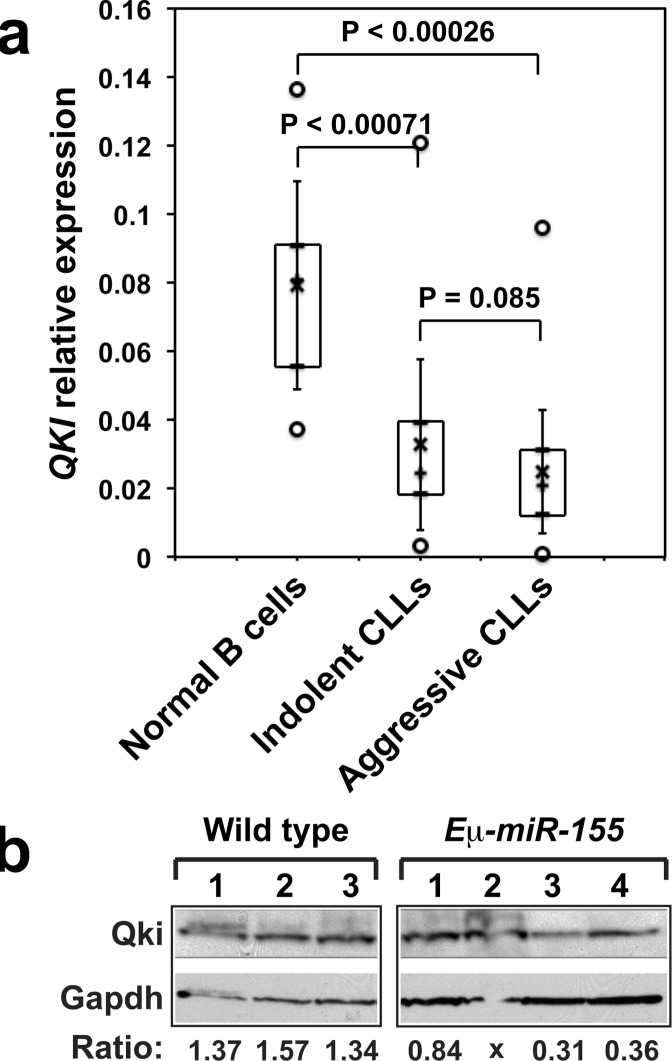
**A.** RNAs extracted from B cells purified from 10 healthy donors (HD), 38 patients with indolent CLL (IND) and 72 patients with aggressive CLL (AGG) were analyzed by qRT-PCR. Boxes include values from the first to the third quartiles; o, extreme data points; +, median; x, mean ± SD. **B.** *Qki* expression in B cells purified from the spleens of wild type or *Eμ-miR-155* transgenic mice was analyzed by Western blotting. Panels are from the same blot. Qki/Gapdh ratios are given under each lane. Spleens from *Eμ-miR-155* mice were: pre-leukemic (1 and 2); and leukemic (3 and 4).

QKI being a target of *miR-155* at least in certain conditions, we would expect its expression to be reduced in leukemias presented with high *miR-155* levels, such as CLL or AML [27–30, 5]. Indeed, the levels of *QKI* transcripts were significantly lower in CLL patients (Figure 6A). In agreement with this result, publicly available array data show that in both, CLL and AML patients, the expression of *QKI* is significantly reduced as compared with healthy donors (data not shown). Finally, Qki levels were also lower in B cells of *E*μ*-miR-155* transgenic mice at the most advanced stage of leukemia (Figure 6B). These results suggest that the disruption of cross-regulations between *QKI, miR-155* and factors implicated in the immune response, may generally be associated with B cell leukemic transformation.

## DISCUSSION

We found that Qki levels are reduced at the onset of immune response to LPS, when *miR-155* levels are on the rise, positioning Qki as an immune factor and a target of *miR-155.* In addition, we found that *QKI* overexpression increases IL-10 production, as well as the total amount of p38, while markedly impairing p38 phosphorylation. It is highly probable that following LPS stimulation, cells sensing the lack of p38 phosphorylation in the presence of excess QKI increase their levels of total p38 to compensate for this deficiency. Of note, K-Ras similarly was previously shown to induce p38 expression but not its phosphorylation, and higher levels of unphosphorylated p38 promote Ras transformation in rat intestinal epithelium through an increased complex formation with Erk-1, -2 kinases [33]. As the reduction of *Qki* levels only took place at the first hours of the immune response, we hypothesize that *miR-155* targeting of *Qki* is restricted only at the beginning of the response, to allow p38 MAPK required activation following LPS challenge. It is thus very likely that during the first hours of LPS signaling, dose-dependent effects take place between Qki, *miR-155* and MAPKs activity, paralleling the oscillatory activity of “early” genes, such as NF-κB and AP-1 activity [34]. Similarly, low *miR-155* expression at the early phase of dendritic cells maturation enables the activation of the p38 pathway, thus favoring IL-1 expression and signaling cascade [35].

Later on, the *miR-155* targeting of *Qki* transcript was impaired. This might be due to the fact that proliferating cells, including human monocytes stimulated with LPS and IFNγ, tend to express mRNAs with shortened 3′-UTR and consequently fewer miRNA target sites [36]. Alternatively, *Qki* 3′-UTR might act as a sponge for *miR-155*. Finally, *miR-155* overexpression in MCF7 breast cancer cells increases the levels of some of its target transcripts through the progressive shortening of their 3′-UTR, including p38 MAPK [37].

Our results show that QKI overexpression increased IL-10 expression and affected p38 MAPK, while *siQKI* modulated the expression of *IRAK3*, a negative regulator of TLR signaling, including p38 MAPK [38]. Altogether, these results suggest that QKI, *miR-155*, p38 and IL-10 [39] are involved in a common regulatory circuitry in B cells and macrophages, and that QKI may represent a new factor of hematopoietic cell transformation. This hypothesis is also supported by previous findings showing that the expression of *QKI* isoforms, changes significantly during hematopoietic differentiation, suggesting for a critical role of QKI in hematopoiesis and function [23].

Our results further show an inverse correlation between *QKI* and *miR-155* expression in CLL and Burkitt's cell lines, and establish that *QKI* might render B cells prone to cell death by increasing Caspase3/7 signaling and *FAS* expression, suggesting that *QKI* may behave as a TSG in B cells. Importantly, *QKI* expression was low in CLL patients, known to have high levels of *miR-155* [27–30], as well as in leukemic B cells isolated from the spleens of *Eμ-miR-155* mice, results further supported by data available in public databases.

Finally, *QKI* unusually long 3′-UTR contains target sites for most miRNAs (targetscan.org). Thus, *QKI* may represents a regulatory hub directing many cellular processes. As human *QKI* 3′-UTR contains three *miR-155* target site, the third one being present only in apes, where QKI came to represent the first most probable target of *miR-155*, one can expect *miR-155-QKI* cross-regulatory interactions to have gained a critical importance in this group.

Mice with spontaneous deletion of *Qki* have marked rapid tremor and seizures, and their entire central nervous system is severely depleted in myelin [40]. As *miR-155* expression is elevated in neuro-inflammatory pathologies such as MS or ALS [41, 42], our data further suggest that a lack of QKI anti-inflammatory input may result in the deleterious dominance of *miR-155* activity, therefore, *miR-155*-QKI interactions could prove to be significantly important for future therapies aimed at neuro-degenerative pathologies presented with high levels of *miR-155*.

In conclusion, while *miR-155* is critical for mounting an effective immune response, its prolonged expression under chronic inflammatory conditions drives immune pathologies and leukemias. Therefore, a better understanding of *miR-155*/QKI functions and their control of expression in immune cells should allow to design new *miR-155*-based cancer immunotherapies. Our findings suggest that when QKI-*miR-155* reciprocal regulation becomes dysfunctional, enhanced *miR-155* activity drives tumor development and evasion of the immune response.

Supplementary information is available at Oncotarget website.

## MATERIALS AND METHODS

Affymetrix microarray (Santa Clara, CA, USA) analyses were submitted to the MIAME database (accession number E-MTAB-2375). Purified CD19^+^ B cells were purchased from Sanguine BioSciences (Sherman Oaks, CA, USA). *Eμ-miR-155* transgenic mice were previously described [6]. B cells from mice were isolated using B-cell purification kit from Miltenyi Biotech (San Diego, CA, USA).

Cells were grown following standard procedures. Information for miRNAs and siRNAs is found in Supplementary Table 1. The fragments of *QKI* 3′UTR and the *Luciferase* reporter construct containing the promoter of *QKI* gene were purchased from SwitchGear. Each *miR-155* site was subsequently mutated using the Quick-Exchange Mutagenesis kit (Agilent; Santa Clara, CA, USA). Human *QKI-5* cDNA was purchased from Genecopeia (Rockville, MD, USA) and recloned into the pCMV-HA expression vector.

Luciferase assays were run 48 hours after transfection as previously described [43]. Caspase-Glo 3/7 kit (Promega; Madison, WI, USA) was used to measure cell death. The assays were performed three times in quadruplicate, and the mean ± S.D. of caspase 3/7 activation (expressed as arbitrary units) is shown.

RNAs were extracted either with TRIzol (Life Technologies) or the RNA purification kit from Norgen (Thorold, ON, Canada). They were subsequently subjected to DNase digestion (Turbo-DNase-Life Technologies). MiRNA and gene qRT-PCRs were respectively performed using the corresponding Assays from Life Technologies (Supplementary Table 1). RT-PCRs were run in triplicates. Values were normalized using one of the following normalizers: *RNU-44, RNU-48, RNU-6B* or *U6* for MiRNA assays and *OAZ1, β-Acti*n or *GAPDH* for Gene Expression assays.

Antibodies and other related information are listed in Supplementary Table 1. Flow cytometry staining was done following standard procedures and run on a Calibur (BD-Biosciences) machine. The data were analyzed using FloJow (Ashland, OR, USA) software. Cytokine production was measured using the ELISA kit (Qiagen) following manufacturer's instruction. Statistical analysis were done using the Student *t* test, and *P* values are provided in the Figure legends.

## SUPPLEMENTARY INFORMATION TABLES


