# Supplementary material for: Quaking and miR-155 interactions in inflammation and leukemogenesis
Source: Oncotarget. 2015 Aug 24;6(28):24599–610. doi: 10.18632/oncotarget.5248 (PMC4694781; doi:10.18632/oncotarget.5248)
Supplement: Supplementary file 1 [file oncotarget-06-24599-s001.pdf]

Quaking and *miR-155* interactions in inflammation and leukemogenesis

Supplementary Information

Supplementary Table 1. Information about reagents used in this study

Catalog number	*Item*	*Manufacturer/Supplier*
Hs00236330_m1	FasL Taqman gene expression assay	Life Technologies
Hs00287641_m1	QKI (probe spans exon 4-5 boundary) Taqman gene expression assay	Life Technologies
Hs01374569_m1	MIR155HG Taqman gene expression assay	Life Technologies
Hs00427923_m1	OAZ1 Taqman gene expression assay	Life Technologies
Mm00498991_m1	Qk (probe spans exon 4-5 boundary) Taqman gene expression assay	Life Technologies
Mm00607939_s1	Actin-β Taqman gene expression assay	Life Technologies
Mm00443258_m1	Tnf Taqman gene expression assay	Life Technologies
Mm01716204_m1	LOC100653389 Taqman gene expression assay	Life Technologies
001094	Control miRNA Assay RNU44	Life Technologies
001006	Control miRNA Assay RNU48	Life Technologies
001093	Control miRNA Assay RNU6B	Life Technologies
002623	Hsa-miR-155 Taqman miRNA assay	Life Technologies
002571	Mmu-miR-155 Taqman miRNA assay	Life Technologies
001973	Control miRNA Assay U6 snRNA	Life Technologies
001093	Control miRNA Assay RNU6B	Life Technologies
PA5-21559	IRAK3 antibody	Life Technologies
9216	Phospho-p38 MAPK (Thr180/Tyr182) (28B10) Mouse	Cell Signaling Technologies
9212	p38 MAPK Antibody	Cell Signaling Technologies
sc-7972	p38 MAPK Antibody	Santa Cruz Biotechnology, Inc.
9106	Phospho-p44/42 MAPK (Erk1/2) (Thr202/Tyr204) (E10) Mouse mAb	Cell Signaling Technologies
9107	p44/42 MAPK (Erk1/2) (3A7) Antibody	Cell Signaling Technologies
9251	Phospho-SAPK/JNK Antibody	Cell Signaling Technologies
9252	SAPK/JNK Antibody	Santa Cruz Biotechnology, Inc.
sc-7345	JNK-2 Antibody	Santa Cruz Biotechnology, Inc.
sc-25336	vinculin (H-10) Antibody	Santa Cruz Biotechnology, Inc.
9099	α-Tubulin (11H10)	Cell Signaling Technologies
ab-3280	Actin beta antibody	Abcam
A300-183A	QKI antibody	Bethyl Laboratories, Inc.
1299001	Stealth siRNA against hQKI HSS114040	Invitrogen/Life technologies
1299001	Stealth siRNA against hQKI HSS114042	Invitrogen/Life technologies
PM13058	Pre-miRmiRNA Precursors mmu-miR-155	Ambion/Life technologies
AM13058	Anti-miR miRNA Inhibitors mmu-miR-155-5p	Ambion/Life technologies
AM17110	Pre-miR™ miRNA Precursor Negative Control #1	Ambion/Life technologies
AM17010	Anti-miR™ miRNA Inhibitor Negative Control #1	Ambion/Life technologies
AM12601	Pre-miRmiRNA Precursors Hsa-miR-155	Ambion/Life technologies
PM12601	Anti-miR miRNA Inhibitors Hsa-miR-155-5p	Ambion/Life technologies
S711421	Promoter QKI	SwitchGear
G8090	Caspase-Glo® 3/7 Assay	Promega
MEM-004A	Mouse Inflammatory Cytokines Multi-Analyte ELISArray™ Kits	SABiosciences /Qiagen

Supplementary Table 2. The hundred most significant target transcripts whose expression in MEC2 CLL cells changed following transfection by siRNAs to *QKI (siQKI*), as determined using the Targetscan program (www.targetscan.org)

Rank	*Transcript symbol*	*Transcript name*	*Predicted* miR-155 *targets*
1	*QKI*	QKI, KH domain containing, RNA binding	+
2	*DHRS2*	Dehydrogenase/reductase (SDR family) member 2	
3	*GNAS*	GNAS complex locus	+
4	*LAMP2*	Lysosomal-associated membrane protein 2	+
5	*DDAH1*	Dimethylarginine dimethylaminohydrolase 1	+
6	*RARA*	Retinoic acid receptor, alpha	
7	*BIVM*	Basic, immunoglobulin-like variable motif containing	
8	*GATM*	Glycine amidinotransferase (L-arginine:glycine amidinotransferase)	+
9	*ADRBK2*	Adrenergic, beta, receptor kinase 2	+
10	*CADM1*	Cell adhesion molecule 1	
11	*CD109*	CD109 molecule	+
12	*VPREB3*	Pre-B lymphocyte 3	
13	*PLEKHB2*	Pleckstrin homology domain containing, family B (evectins) member 2	+
14	*CCDC50*	Coiled-coil domain containing 50	+
15	*ARHGAP32*	Rho GTPase activating protein 32	+
16	*IFI44*	Interferon-induced protein 44	
17	*AIF1*	Allograft inflammatory factor 1	
18	*PHTF2*	Putative homeodomain transcription factor 2	
19	*ICK*	Intestinal cell (MAK-like) kinase	
20	*CCDC88A*	Coiled-coil domain containing 88A	
21	*ADAM17*	ADAM metallopeptidase domain 17	
22	*SOS1*	Son of sevenless homolog 1 (Drosophila)	+
23	*ZC3HAV1L*	Zinc finger CCCH-type, antiviral 1-like	
24	*PTPN3*	Protein tyrosine phosphatase, non-receptor type 3	
25	*SRGAP2C*	SLIT-ROBO Rho GTPase activating protein 2C	
26	*NEDD4*	Neural precursor cell expressed, developmentally down-regulated 4, E3 ubiquitin protein ligase	
27	*MAPT*	Microtubule-associated protein tau	
28	*FCRLA*	Fc receptor-like A	+
29	*CD80*	CD80 molecule	
30	*RNF125*	Ring finger protein 125, E3 ubiquitin protein ligase	
31	*FAM208A*	Family with sequence similarity 208, member A	
32	*FAM217B*	Family with sequence similarity 217, member B	+
33	*EML4*	Echinoderm microtubule associated protein like 4	
34	*ZNF785*	Zinc finger protein 785	
35	*MCAM*	Melanoma cell adhesion molecule	
36	*P2RY10*	Purinergic receptor P2Y, G-protein coupled, 10	

37	*FAF1*	Fas (TNFRSF6) associated factor 1	
38	*NIPSNAP3B*	Nipsnap homolog 3B (C. elegans)	
39	*LRP11*	Low density lipoprotein receptor-related protein 11	
40	*IGF1*	Insulin-like growth factor 1 (somatomedin C)	+
41	*WDR41*	WD repeat domain 41	
42	*PLAUR*	Plasminogen activator, urokinase receptor	
43	*PHF10*	PHD finger protein 10	
44	*MACC1*	Metastasis associated in colon cancer 1	+
45	*SP100*	SP100 nuclear antigen	
46	*RMND5A*	Required for meiotic nuclear division 5 homolog A (S. cerevisiae)	
47	*RSF1*	Remodeling and spacing factor 1	
48	*YME1L1*	YME1-like 1	+
49	*ITPR1*	Inositol 1,4,5-trisphosphate receptor, type 1	
50	*WDR27*	WD repeat domain 27	
51	*RASSF4*	Ras association (RalGDS/AF-6) domain family member 4	
52	*RALGPS2*	Ral GEF with PH domain and SH3 binding motif 2	
53	*GREB1*	Growth regulation by estrogen in breast cancer 1	
54	*PNMA2*	Paraneoplastic Ma antigen 2	
55	*KCNJ11*	Potassium inwardly-rectifying channel, subfamily J, member 11	
56	*NFIC*	Nuclear factor I/C (CCAAT-binding transcription factor)	
57	*SSBP3*	Single stranded DNA binding protein 3	
58	*SCN3A*	Sodium channel, voltage-gated, type III, alpha subunit	
59	*ACVR2B*	Activin A receptor, type IIB	+
60	*MAP9*	Microtubule-associated protein 9	+
61	*PLEKHG4*	Pleckstrin homology domain containing, family G (with RhoGef domain) member 4	
62	*FAM49A*	Family with sequence similarity 49, member A	
63	*TBC1D30*	TBC1 domain family, member 30	+
64	*TWF1*	Twinfilin, actin-binding protein, homolog 1 (Drosophila)	+
65	*ABAT*	4-aminobutyrate aminotransferase	+
66	*CSDA*	Cold shock domain protein A	
67	*SLA*	Src-like-adaptor	+
68	*ATP7B*	ATPase, Cu++ transporting, beta polypeptide	
69	*YES1*	v-yes-1 Yamaguchi sarcoma viral oncogene homolog 1	+
70	*GDA*	Guanine deaminase	
71	*LOXL2*	Lysyl oxidase-like 2	
72	*IGF2BP1*	Insulin-like growth factor 2 mRNA binding protein 1	
73	*TBRG1*	Transforming growth factor beta regulator 1	+
74	*RLN2*	Relaxin 2	
75	*PICALM*	Phosphatidylinositol binding clathrin assembly protein	+
76	*JAG1*	Jagged 1	
77	*TMX4*	Thioredoxin-related transmembrane protein 4	
78	*PXDN*	Peroxidasin homolog (Drosophila)	
79	*SIGLEC10*	Sialic acid binding Ig-like lectin 10	

80	*AKD1*	Adenylate kinase domain containing 1	
81	*CCR10*	Chemokine (C-C motif) receptor 10	
82	*KMO*	Kynurenine 3-monooxygenase (kynurenine 3-hydroxylase)	
83	*RASGRF1*	Ras protein-specific guanine nucleotide-releasing factor 1	
84	*CTBP2*	C-terminal binding protein 2	
85	*PAG1*	Phosphoprotein associated with glycosphingolipid microdomains 1	+
86	*PITPNC1*	Phosphatidylinositol transfer protein, cytoplasmic 1	
87	*TDRD12*	Tudor domain containing 12	
88	*IRAK3*	Interleukin-1 receptor-associated kinase 3	
89	*CDC42SE2*	CDC42 small effector 2	+
90	*CXCR5*	Chemokine (C-X-C motif) receptor 5	
91	*C7orf41*	Chromosome 7 open reading frame 41	+
92	*ST3GAL6*	ST3 beta-galactoside alpha-2,3-sialyltransferase 6	
93	*TWSG1*	Twisted gastrulation homolog 1 (Drosophila)	+
94	*TUFT1*	Tuftelin 1	
95	*SLC1A1*	Solute carrier family 1 (neuronal/epithelial high affinity glutamate transporter, system Xag), member 1	
96	*SRSF6*	Serine/arginine-rich splicing factor 6	
97	*MCOLN3*	Mucolipin 3	
98	*LY9*	Lymphocyte antigen 9	
99	*CNFN*	Cornifelin	
100	*SORL1*	Sortilin-related receptor, L (DLR class) A repeats containing	+

Supplementary Table 3. The hundred most significant target transcripts whose expression in BJAB Burkitt's lymphoma cells changed following transfection by siRNAs to *QKI (siQKI)*, as determined using the Targetscan program (www.targetscan.org)

Rank	*Transcript symbol*	*Transcript name*	*Predicted* miR-155 *targets*
1	*QKI*	QKI, KH domain containing, RNA binding	+
2	*GNAS*	GNAS complex locus	+
3	*CCDC88A*	Coiled-coil domain containing 88A	
4	*CCR7*	Chemokine (C-C motif) receptor 7	
5	*GALNT12*	UDP-N-acetyl-alpha-D-galactosamine:polypeptide N-acetylgalactosaminyltransferase 12 (GalNAc-T12)	+
6	*ZNF232*	Zinc finger protein 232	
7	*DHRS9*	Dehydrogenase/reductase (SDR family) member 9	
8	*GJA3*	Gap junction protein, alpha 3, 46kDa	
9	*TFEC*	Transcription factor EC	+
10	*H2AFY*	H2A histone family, member Y	
11	*SLC25A13*	Solute carrier family 25 (aspartate/glutamate carrier), member 13	
12	*LTB*	Lymphotoxin beta (TNF superfamily, member 3)	
13	*GCLM*	Glutamate-cysteine ligase, modifier subunit	
14	*EGR1*	Early growth response 1	
15	*CAMSAP2*	Calmodulin regulated spectrin-associated protein family, member 2	
16	*ID3*	Inhibitor of DNA binding 3, dominant negative helix-loop-helix protein	
17	*TXLNB*	Taxilin beta	+
18	*LRRC61*	Leucine rich repeat containing 61	
19	*NTNG1*	Netrin G1	
20	*CCDC74B*	Coiled-coil domain containing 74B	
21	*FSD1L*	Fibronectin type III and SPRY domain containing 1-like	
22	*MLH3*	MutL homolog 3 (E. coli)	
23	*RALGPS2*	Ral GEF with PH domain and SH3 binding motif 2	
24	*ACPP*	Acid phosphatase, prostate	
25	*ERAP2*	Endoplasmic reticulum aminopeptidase 2	
26	*DHRS2*	Dehydrogenase/reductase (SDR family) member 2	
27	*TSEN15*	TRNA splicing endonuclease 15 homolog (S. cerevisiae)	
28	*SATB1*	SATB homeobox 1	+
29	*PIK3CG*	Phosphatidylinositol-4,5-bisphosphate 3-kinase, catalytic subunit gamma	
30	*VASH2*	Vasohibin 2	
31	*MYLIP*	Myosin regulatory light chain interacting protein	
32	*GREB1*	Growth regulation by estrogen in breast cancer 1	
33	*SLC6A16*	Solute carrier family 6, member 16	
34	*HSPA13*	Heat shock protein 70kDa family, member 13	
35	*CYP1B1*	Cytochrome P450, family 1, subfamily B, polypeptide 1	
36	*CD38*	CD38 molecule	+
37	*TGFBR1*	Transforming growth factor, beta receptor 1	+

38	*DCN*	Decorin	
39	*VCAM1*	Vascular cell adhesion molecule 1	
40	*FCRLA*	Fc receptor-like A	+
41	*RAC1*	Ras-related C3 botulinum toxin substrate 1 (rho family, small GTP binding protein Rac1)	+
42	*CMPK2*	Cytidine monophosphate (UMP-CMP) kinase 2, mitochondrial	+
43	*CARKD*	Carbohydrate kinase domain containing	
44	*PEA15*	Phosphoprotein enriched in astrocytes 15	+
45	*PICALM*	Phosphatidylinositol binding clathrin assembly protein	+
46	*ZEB2*	Zinc finger E-box binding homeobox 2	
47	*TOB1*	Transducer of ERBB2, 1	
48	*LAMP3*	Lysosomal-associated membrane protein 3	
49	*RNF144A*	Ring finger protein 144A	
50	*IL1B*	Interleukin 1, beta	
51	*CD69*	CD69 molecule	
52	*TMEM123*	Transmembrane protein 123	+
53	*FOXA3*	Forkhead box A3	
54	*CPA6*	Carboxypeptidase A6	
55	*LAMP2*	Lysosomal-associated membrane protein 2	+
56	*LAPTM4B*	Lysosomal protein transmembrane 4 beta	
57	*KCTD15*	Potassium channel tetramerisation domain containing 15	
58	*ARL1*	ADP-ribosylation factor-like 1	+
59	*KL*	Klotho	
60	*AFF1*	AF4/FMR2 family, member 1	+
61	*SUSD1*	Sushi domain containing 1	
62	*KCNJ3*	Potassium inwardly-rectifying channel, subfamily J, member 3	+
63	*TMX4*	Thioredoxin-related transmembrane protein 4	
64	*RNF170*	Ring finger protein 170	
65	*CSRNP3*	Cysteine-serine-rich nuclear protein 3	
66	*SLC28A3*	Solute carrier family 28 (sodium-coupled nucleoside transporter), member 3	
67	*C11orf54*	Chromosome 11 open reading frame 54	
68	*BTNL9*	Butyrophilin-like 9	
69	*LZTS1*	Leucine zipper, putative tumor suppressor 1	
70	*WDR76*	WD repeat domain 76	
71	*TIMP1*	TIMP metallopeptidase inhibitor 1	
72	*USP6NL*	USP6 N-terminal like	
73	*IL32*	Interleukin 32	
74	*FSCN1*	Fascin homolog 1, actin-bundling protein (Strongylocentrotus purpuratus)	
75	*ERMP1*	Endoplasmic reticulum metallopeptidase 1	+
76	*DYX1C1*	Dyslexia susceptibility 1 candidate 1	
77	*GPNMB*	Glycoprotein (transmembrane) nmb	
78	*IRS1*	Insulin receptor substrate 1	
79	*KLF9*	Kruppel-like factor 9	

80	*SERPINA9*	Serpin peptidase inhibitor, clade A (alpha-1 antiproteinase, antitrypsin), member 9	
81	*KIAA2022*	KIAA2022	+
82	*PTPDC1*	Protein tyrosine phosphatase domain containing 1	
83	*EPB41L4B*	Erythrocyte membrane protein band 4.1 like 4B	+
84	*ZNF426*	Zinc finger protein 426	
85	*SOAT1*	Sterol O-acyltransferase 1	
86	*TMEM63C*	Transmembrane protein 63C	
87	*HSF2*	Heat shock transcription factor 2	
88	*LRRC33*	Leucine rich repeat containing 33	
89	*MYL12A*	Myosin, light chain 12A, regulatory, non-sarcomeric	
90	*PRDM13*	PR domain containing 13	
91	*MYO1B*	Myosin IB	+
92	*ASAP3*	ArfGAP with SH3 domain, ankyrin repeat and PH domain 3	
93	*TOX2*	TOX high mobility group box family member 2	
94	*ZNF268*	Zinc finger protein 268	+
95	*MYO7B*	Myosin VIIB	
96	*FCRL3*	Fc receptor-like 3	
97	*SAMD9L*	Sterile alpha motif domain containing 9-like	
98	*MACROD2*	MACRO domain containing 2	
99	*EVL*	Enah/Vasp-like	
100	*SEMA4C*	Sema domain, immunoglobulin domain (Ig), transmembrane domain (TM) and short cytoplasmic domain, (semaphorin) 4C	
